# Myocardial injury defined as elevated high-sensitivity cardiac troponin T is associated with higher mortality in patients seeking care at emergency departments with acute dyspnea

**DOI:** 10.1186/s12873-023-00787-w

**Published:** 2023-04-05

**Authors:** T Wessman, A Zorlak, Per Wändell, O Melander, AC Carlsson, T Ruge

**Affiliations:** 1 Department of Emergency and Internal Medicine, Skåne University Hospital, Malmö, Sweden; 2 Department of Clinical Sciences Malmö, Department of Internal Medicine, Lund University, Skåne University Hospital, Malmö, Sweden; 3 Department of Neurobiology, Care Sciences and Society, Karolinska Institutet, Huddinge, Sweden; 4 Academic Primary Care Center, Region Stockholm, Sweden

**Keywords:** Congestive heart failure, Emergency department, High-sensitivity cardiac troponin T, Myocardial injury

## Abstract

**Background:**

Elevated levels of cardiac troponin T has been observed in patients seeking care at the emergency department (ED) presenting with chest pain but without myocardial infarction (MI). The clinical importance of this observation remains, however, still unclear. Our main aim was to study the role of cardiac troponin T in patients admitted to the emergency department with acute dyspnea, a group of patients with a high cardiovascular comorbidity, but no primary acute MI.

**Population and methods:**

Patients from the age of 18 seeking care at the ED for dyspnea, without an acute cardiac syndrome, and with a recorded assessment of high-sensitivity cardiac troponin T (hs-cTnT), were included (n = 1001). Patients were categorized into 3 groups by hs-cTnT level, i.e. <15, 15–100 and > 100 µg/l. Cox regression with Hazard Ratios (HRs) and 95% Confidence Intervals (CI) for 3-months mortality was performed, with adjustment for sex, age, respiratory frequency, saturation, CHF, renal disease, and BMI.

**Results:**

Fully adjusted HRs (95% CI) for 3-month mortality, with hs-cTnT < 15 µg/l as reference level, showed for hs-cTnT 15–100 a HR of 3.682 (1.729–7.844), and for hs-cTnT > 100 a HR of 10.523 (4.465–24.803).

**Conclusion:**

Elevated hs-cTnT seems to be a relevant marker of poor prognosis in patients with acute dyspnea without MI and warrants further validation and clinical testing.

**Supplementary Information:**

The online version contains supplementary material available at 10.1186/s12873-023-00787-w.

## Introduction

Acute dyspnea is one of the most common and challenging presentations at the emergency department [1, 2]. An array of patient categories, presenting with different comorbidities, age-groups, severity, risk, and prognosis seek care with acute dyspnea as the main symptom. Therefore, a quick and safe identification of patients at risk for worse prognosis is of particular importance [1].

High-sensitivity cardiac troponin T (hs-cTnT) has been found to safely rule out myocardial infarction, in a Swedish study within one hour [3], and according to a review within 4 h at emergency department [4]. On the other hand, hs-cTnT has been evaluated in patients seeking care for chest pain in absence of myocardial infarction, showing that high hs-cTnT values can mirror myocardial injury in other types of myocardial injuries than MI, reflecting the complex biology of the cardiac sarcomeres of the cardiac myocytes [5–8].

In patients with a myocardial infarction, dyspnea is a common symptom [9]. In fact, the clinically used level of hs-cTnT as a sign of myocardial injury, has been associated with both short- and long-term mortality in patients presenting with dyspnea [10]. However, hs-cTnT may be influenced by other cardiac conditions, including heart failure, myocarditis, arrhythmias, and aortic dissections, as well as of non-cardiac factors, such as male gender, high age, obesity, stroke, and renal impairment [11].

A study examining patient characteristics and its association with myocardial injury mirrored by hs-cTnT in patients with dyspnea seeking care would be of clinical importance, as identifying risk factors for myocardial injury could facilitate the triaging and management of these patients. We therefore designed a study, focusing on patients presenting with dyspnea, examining the association between myocardial injury and mortality but in the absence of acute myocardial infarction, with the aim to explore the association between myocardial injury and patient characteristics: age, sex, certain cardiovascular comorbidities, and symptom presentation.

We hypothesized that elevated levels of hs-cTnT in patients seeking the ED with acute dyspnea was associated with increased mortality risk as well that these patients were of older age, had a higher cardiovascular comorbidity and were identified with higher medical urgency at the ED.

## Method

### Population and methods

**Fig. 1:**
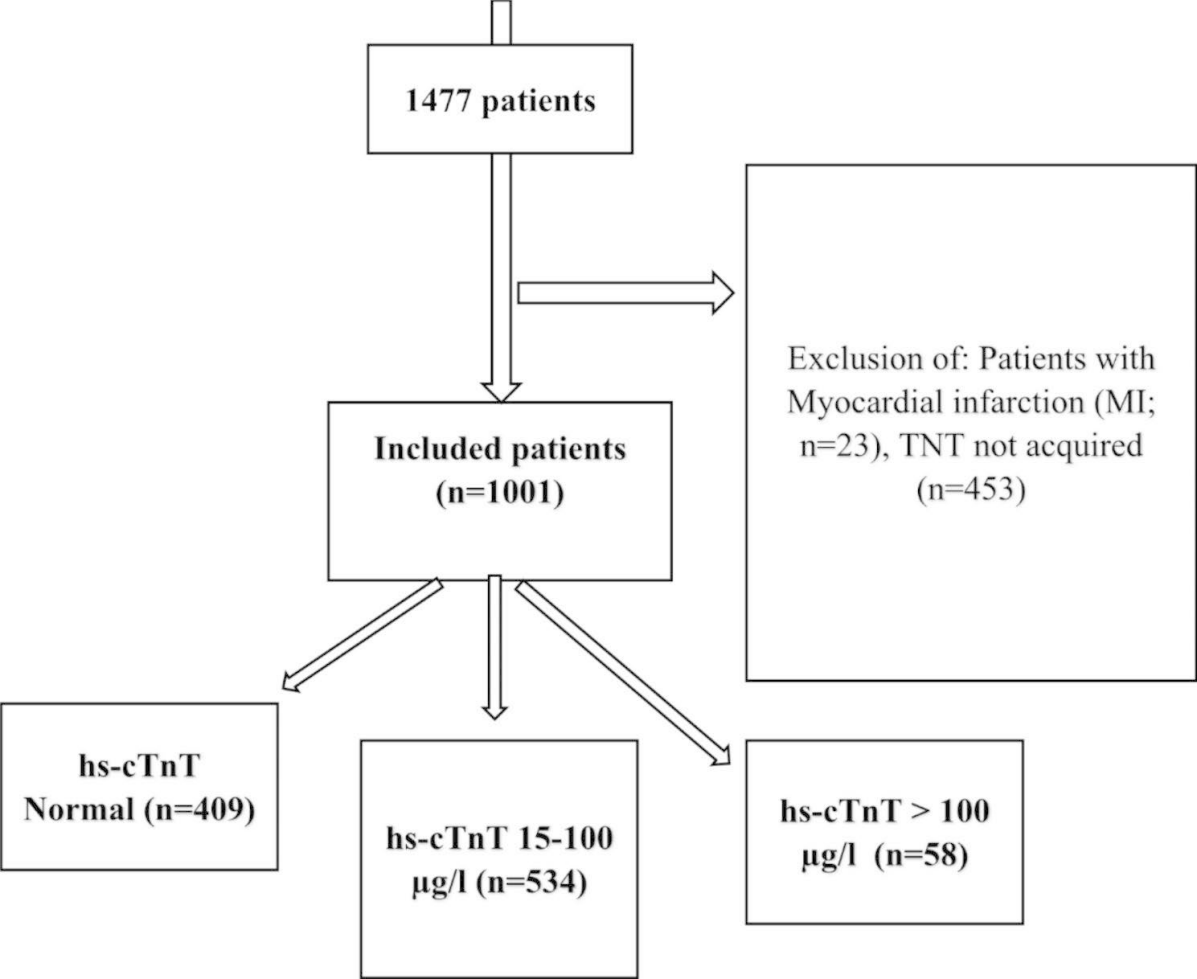
Flow chart of exclusion and inclusion of patients, and categorisation by high-sensitive cardiac troponin T (hs-TnT)

Data was obtained from the Acute Dyspnea Study (ADYS) cohort, which was collected from December 13th 2013 to January15th 2019. Patients from the age of 18 seeking care at the emergency department (ED) for dyspnea, were included. The inclusion took place Mondays through Fridays between 8.00 AM and 5.00 PM at the ED of Skåne University Hospital, Malmö. Participants were triaged according to Medical Emergency Triage and Treatment System-Adult score (METTS-A) [12, 13]. Patients were not included if they were in critical condition or obtunded. In the present study 1477 unique patients were included in the ADYS study until December 31 2017. Out of the 1477 patients in the ADYS database, 453 patients were due to missing TNT values and 23 were excluded due to events of myocardial infarction making the final cohort n = 1001 patients (Fig. 1).

After inclusion, all included patients were interviewed by a research nurse about their health, medications, symptoms, and social situation according to a standardized and approved questionnaire.

Patients were questioned regarding smoking habits and categorized as non-smokers, former smokers (cessation one month ago or longer), or active smokers (regularly smoking the past month or longer), disease history associated with dyspnea (i.e. congestive heart failure, chronic obstructive pulmonary disease, asthma, coronary artery disease, atrial fibrillation, restrictive lung disease, cancer, thromboembolic disease, or rheumatic disease) and current medications. After inclusion, the research nurses again reviewed the patient journals in order to confirm the details, with the support of senior physicians whenever uncertainties occurred. If the nurses here observed diagnoses compatible with a primary cardiac diagnosis the patients were excluded.

Originally METTS-A uses five clinical priority levels with increasing clinical priority: blue (lowest clinical priority - not life-threatening), green, yellow, orange, and red (highest clinical priority - life-threatening). The lowest clinical priority level blue was not used in the clinical triage of the patients included here due to the local triage routines, as the lowest clinical triage priority here was green. The level of dyspnea was graded by the research nurses on all patients i.e. even patients not suffering from CHF, according to the New York Heart Association functional classification of heart failure, i.e. levels I-IV [14].

Patient comorbidity was here defined as having a history of hypertension, renal disease, coronary heart disease (CHD), congestive heart failure (CHF) and atrial fibrillation (AF).

Patient collection of Hs-cTnT was based on triage criteria according to METTS-A or if the treating physician found it necessary based on patient symptom presentation or clinical suspicion. According to METTS-A triage system troponin sampling is advised at certain levels of syntax related symptom severity, in this case for the algorithm “Dyspnea” troponin sampling was mandatory for patients with a clinical severity score of yellow or higher. Only the first hs-cTnT taken at the emergency department was registered in the ADYS database. Myocardial injury in the present study was defined as increased troponin, i.e. hs-cTnT over 15 µg/l [15, 16].

Data was received from Swedish National Board of Health and Welfare regarding time of death and the ICD codes registered in the national database. Mortality data were acquired up to December 31st 2017.

### Statistics

Categorial variables were displayed as frequencies, and statistically tested with Pearson’s Chi Square test. Continuous variables were displayed as means (and standard deviations, sd), or medians (and interquartile range, IQR). For comparison between two groups Mann Whitney’s U-test was used, as all distributions were skewed. When multiple groups and non-normal distribution, Kruskal Wallis 1-way ANOVA was used.

Cox regression with Hazard Ratios (HRs) and 95% confidence interval (95% CI) was used to estimate the relative risk for mortality at 30-days. Model A, partially adjusted, tested each separate variables with adjustment for age and gender. Model B, a multivariate model tested all the variables significant in Model A with adjustment for age and gender. In the Cox regression analysis statistical significance was set at p < 0.01, due to multiple testing. Statistical significance level was otherwise set at p < 0.05. Statistical analysis was done in SPSS version 25 (SPSS Inc, Chicago, IL).

## Results

Patients (Table 1) were subdivided by levels of hs-cTnT with categorization into three groups based on their hs-cTnT levels (Table 2). Compared to patients with normal hs-cTnT, patients with increased hs-cTnT, were older, were more often males, more often hospitalized, had more deranged vital signs at admittance, were admitted with more severe symptoms, had fewer normal ECGs, a higher proportion of ECGs with ST-depression. Additionally, patients with increased hs-cTnT levels had a far higher proportion of comorbidities and a higher mortality after 3-months follow-up. As regards hs-cTnT in men and women, men had higher values, mean 44.84 ng/l (sd 84.69) and median 27 ng/l (IQR 13–50) vs. in women 32.38 ng/l (sd 121.30) and 14 ng/l (IQR 6–28), respectively (p < 0.003). Compared to patients without acquired hs-cTnT patients with acquired hs-cTnT were older, were more often men, were admitted with more severe symptoms, had less normal ECGs and a higher proportion of comorbidities compared to patients without acquired hs-cTnT (Supplementary Table 1).

**Table 1:** Characteristics in patients seeking care at the emergency department with dyspnea by levels of high-sensitive cardiac troponin-T (hs-cTnT) values

Variable	hs-cTnT < 15 ng/l	hs-cTnT 15–100 ng/l	hs-cTnT > 100 ng/l	p-value
hs-cTnT, ng/L (mean +/- SD)	7.79 +/-3.16	36.66 +/-20.26	267.26 +/-364.06	
hs-cTnT, ng/L (median, IQR)	7.00 (5.0–11.00)	30.00 (21.00–45.00)	148.50 (101.00–233.50)	
Age, years (mean, +/- SD)	60.3 +/-19.0	78.5 +/- 11.1	78.1 +/-12.0	< 0.0001*
Age, years (median, IQR)	63.6 (45.5–75.1)	79.7 (71.8–87.1)	81.1 (71.5–86.9)	
Gender man (N, %)	133 (32.5%)	300 (56.2%)	37 (63.8%)	< 0.001**
History of CAD (N, %)	55 (13.4%)	226 (42.3%)	35 (60.3%)	< 0.001**
History of CHF (N, %)	50 (12.2%)	281 (52.6%)	32 (55.2%)	< 0.001**
History of AFIB (N, %)	69 (16.9%)	226 (42.3%)	23 (39.7%)	< 0.001**
History of hypertension (N, %)	119 (29.1%)	286 (53.6%)	286 (53.6%)	< 0.001**
History of renal disease (N, %)	6 (1.5%)	69 (12.9%)	20 (34.5%)	< 0.001**
METTS-green (N, %)	25 (6.1%)	16 (3.0%)	1 (1.7%)	< 0.001**
METTS-yellow (N, %)	261 (63.8%)	215 (40.3%)	13 (22.4%)	
METTS-orange (N, %)	102 (24.9%)	208 (39.0%)	24 (41.4%)	
METTS-red (N, %)	19 (4.6%)	94 (17.6%)	19 (32.8%)	
NYHA-class I (%)	204 (49.9%)	93 (17.4%)	5 (8.6%)	< 0.001**
NYHA-class II (%)	116 (28.4%)	183 (34.3%)	17 (29.3%)	
NYHA-class III (%)	48 (11.7%)	108 (20.2%)	12 (20.7%)	
NYHA-class IV (%)	36 (8.8%)	144 (27.0%)	24 (41.4%)	
ECG normal ST (N, %)	263 (64.3%)	180 (33.7%)	12 (20.7%)	< 0.001**
ECG ST Elevation (N, %)	2 (0.5%)	8 (1.5%)	3 (5.2%)	0.011** 0.008***
ECG ST Depression (N, %)	24 (5.9%)	69 (12.9%)	15 (25.9%)	< 0.001**
ECG Abnormal T-wave (N,%)	23 (5.6%)	45 (8.4%)	9 (15.5%)	0.020** 0.008***
Ambulans (N, %)	130 (31.8%)	375 (70.2%)	46 (79.3%)	< 0.001**
Alarm (N, %)	17 (4.2%)	71 (13.3%)	19 (32.8%)	< 0.001**
Hospitalization (%)	130 (31.8%)	412 (77.2%)	56 (96.6%)	< 0.001**
Mortality 3-months (%)	9 (2.2%)	88 (16.5%)	24 (41.4%)	< 0.001**
Saturation % (mean +/-SD)	95.9 +/-4.0	91.1 +/-9.3	88.7 +/-8.8	< 0.0001*
Saturation % (median, IQR)	97 (94– 99)	93 (89–97)	91 (84–96)	
Respiratory frequency (mean, +/- SD)	21.7 +/-7.1	26.0 +/-7.7	28.0 +/-7.8	< 0.0001*
Respiratory frequency (median, IQR)	20 (18– 24)	24 (20– 29)	26 (22– 35)	
BMI kg/m^2^ (mean +/-SD)	26.8 +/-5.5	27.0 +/-6.6	25.9 +/-6.7	0.23*
BMI kg/m^2^ (median, IQR)	26.0 (23.0– 30.4)	26.0 (22.9–30.3)	24.6 (21.8–27.9)	

*Asymptotic 2 side Kruskal_Wallis; **Pearson Chi^2^; *** linear-by linear

CAD: coronary artery disease; CHF: congestive heart failure; AF: atrial fibrillation; ED: emergency department

IQR: interquartile range

**Table 2:** The Hazard Ratios (HRs) by Cox regression models for separate variables with adjustment for age and gender for 3-months mortality by high-sensitive cardiac troponin-T (hs-cTnT) levels in patients seeking emergency care due to dyspnea

3 months mortality	HR	95% CI	*p-*value
Troponin < 15 ng/L (n = 409)	ref		
Troponin **15–100 ng/L** (n = 534)	**5.04**	**2.44–10.42**	**< 0.001**
Troponin **> 100 ng/L** (n = 58)	**16.35**	**7.29–36.64**	**< 0.001**
METTS green	ref		
METTS yellow	1.83	0.44–7.56	0.402
METTS orange	2.30	0.56–9.49	0.250
METTS red	3.62	0.86–15.20	0.079
Ever smoker	0.87	0.59–1.28	0.478
EKG ST-level			
1 = normal	ref		
2 = ST elevation	2.30	0.71–7.43	0.17
3 = ST depression	1.22	0.69–2.16	0.50
4 = patological T-wave	1.27	0.65–2.48	0.49
5 = other changes	1.08	0.69–1.68	0.74
9 = unknown	2.55	0.79–8.24	0.12
**Respiratory frequency (per min)**	**1.02**	**1.00–1.04**	**0.041**
**Saturation (%)**	**0.98**	**0.97–0.99**	**< 0.001**
CAD	0.73	0.54–1.13	0.19
**CHF**	**0.57**	**0.39–0.83**	**0.003**
AF	1.02	0.76–1.36	0.92
Hypertension	1.08	0.75–1.55	0.68
**Renal disease**	**0.53**	**0.33–0.84**	**0.007**
**BMI (kg/m** ^**2**^ **)**	**0.95**	**0.92–0.99**	**0.007**

Significant factors marked in bold

As we compared the two groups with an increase in hs-cTnT above clinical cut off (hs-cTnT 15–100 vs. hs-cTnT > 100) we found a significant differences in hospitalization rate (77.6% vs. 93.9%), proportions of “high medical urgency cases “METTS-A red” (17.5% vs 30.6%), proportions of patients with renal disease (12.5% vs 36.7%) and in 3 months mortality (16.6% vs 40.8%).

**Fig. 2:**
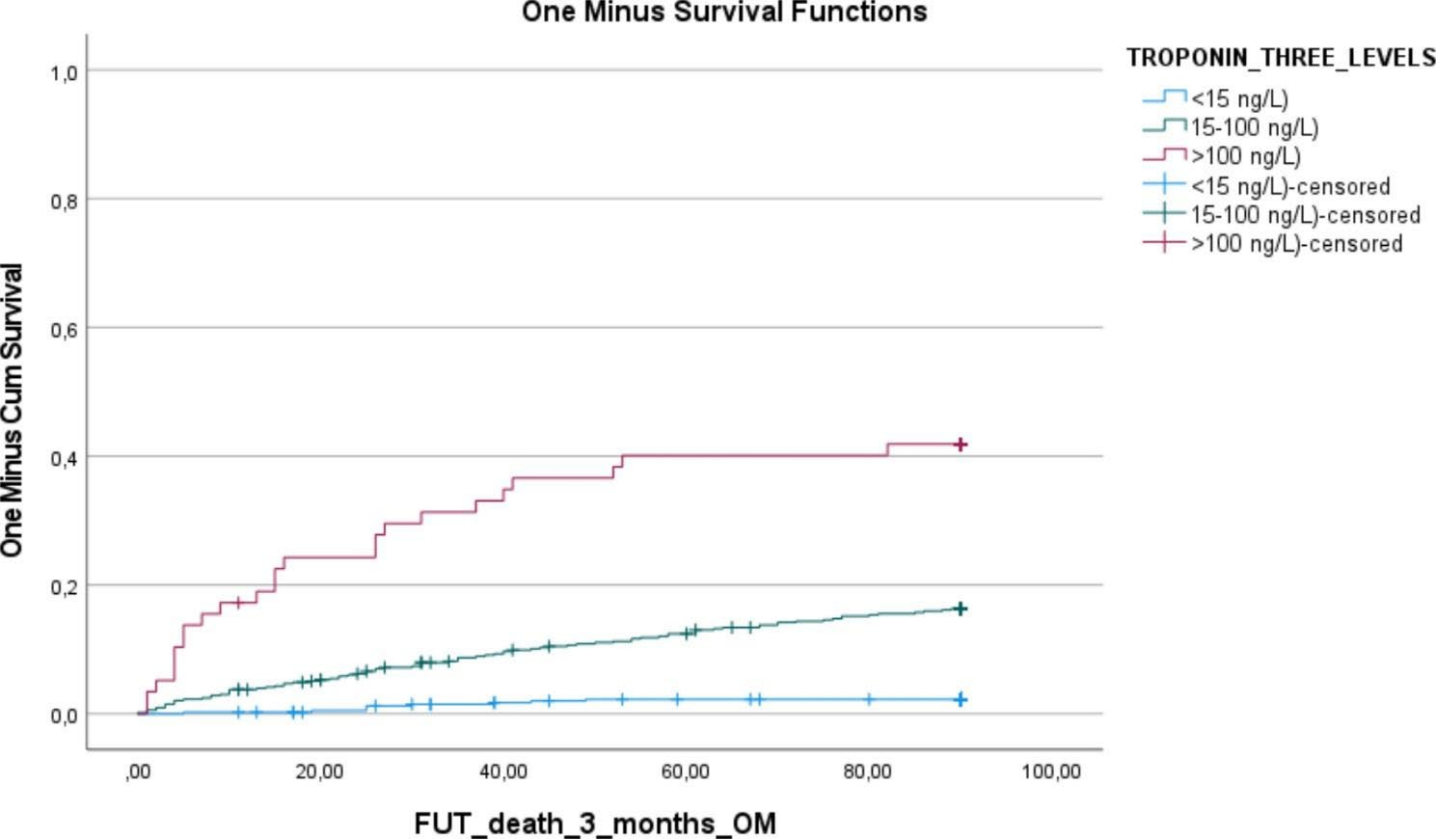
Kaplan-Meier curve for the three groups

Cox regression analysis for 3-months mortality for the different factors by the three groups of hs-cTnT values with adjustment for age and sex is shown in Table 3. Five factors were significantly associated with mortality, i.e. respiratory frequency, saturation, CHF, renal disease, and BMI. Table 3 shows the result of the multivariate model, with HRs for the hs-cTnT 15–100 group being 3.682, and for the hs-cTnT > 100 10.523. Kaplan-Meier curve is shown in Fig. 2.

**Table 3:** Multivariate model with Hazard Ratios (HRs) by Cox regression (n = 1001) with adjustment for age and gender, and significant factors from single models partly adjusted for age and gender included, i.e. troponin categorical, respiratory rate, saturation, CHF, renal disease and BMI level

3 months mortality	HR	95% CI	P value
Age	0.85	0.58–1.26	0.42
**Gender**	**1.02**	**1.01–1.04**	**0.014**
Troponin < 15 ng/L	**ref**		
**Troponin 15–100 ng/L**	**3.68**	**1.73–7.84**	**< 0.001**
**Troponin > 100 ng/L**	**10.52**	**4.47–24.80**	**< 0.001**
Respiratory frequency (per min)	1.01	0.98–1.03	0.63
**Saturation (%)**	**0.98**	**0.97–1.00**	**0.034**
CHF	0.73	0.49–1.08	0.12
Renal disease	0.77	0.53–1.14	0.19
**BMI (kg/m** ^**2**^ **)**	**0.95**	**0.91–0.98**	**0.005**

Significant factors marked in bold

The mean and median values of the hs-cTnT values for patients who died within 3 months vs. those who survived were for mean values 99.24 ng/l (sd 247.70) vs. 29.87 ng/l (sd 61.54), respectively, and for median values 44 ng/l (IQR 26–83) vs. 17 ng/l (IQR 7–32), respectively, and as distributions were skewed Mann-Whitney’s U-test was used (p < 0.001).

## Discussion

The main results showed a significantly higher 3-months mortality for higher hs-cTnT. Patients with higher hs-cTnT levels were significantly older, had significantly more comorbidities, presented with more severe symptoms, had significantly more deranged vital signs, fewer normal ECGs, and more ECGs with ST depression. Correlation analysis showed associations between elevated levels of hs-cTnT and age > 75 years, congestive heart failure and incidence of comorbidities.

According to clinical guidelines the definition of a primary acute MI is based on typical presenting symptoms, electrocardiographic findings and by a rise or fall in the troponin level above the 99th percentile [15]. This algorithm has been proven to be clinically effective in identifying patients with acute cardiac damage and is so far still regarded as the golden standard. The major role for the interpretation of hs-cTNT levels in typical patients is to rule in or rule out acute MI [17].

With the clinical implementation of hs-cTNT it has been clear that circulating levels of hs-cTNT are frequently increased above the 99th percentile in patients without signs of acute coronary events. Levels of hs-cTNT are higher in male patients, are increased in aged patients, are associated with the number of comorbidities, are increased in systemic inflammatory diseases and in patients with decreased glomerular filtration rate and finally are increased in patients with left ventricular dysfunction as well as in patients with atrial flutter [7, 18]. A study on 20 000 in and outpatients undergoing blood testing for any clinical reason showed that the 99 the percentile of hs-cTNT for the whole population was 189 ng/L when patients with acute coronary events were excluded. In ED specific patients the number was 215 ng/L [19].

Importantly, hs-cTNT levels have previously been analyzed in patients with chest pain without myocardial infarction at the emergency department, showing that these patients often have myocardial injury and lower GFR [7, 8]. It has actually been suggested that elevated hs-cTnT levels at the emergency department in patients with no myocardial infarction should be investigated routinely and at least treated with antiplatelet drugs, as these patients often have a reduced left ventricular ejection fraction [8]. In summary, interpretation of elevated hs-cTNT levels have to be performed with care at the ED as well as in other indications of testing. Elevated hs-cTNT levels in other states than acute coronary diseases could be associated with cardiac injury and could thus be important predictors of cardiovascular mortality and morbidity [20]. However, unlike patients with myocardial infarction, there is little guidance to be found in how to treat and follow up patients with elevated hs-cTnT and no myocardial infarction and no evidence exists that patients with increased hs-cTnT would need urgent management for ischemic disease [7].

In comparison to the literature, our data confirm the previously shown associations between hs-cTNT and level of comorbidity [21] and increased age [22]. Additionally, we show that these patients, when admitted to the ED for acute dyspnea also had fewer normal ECGs. We also show that significant proportions of our patients display specific comorbidities such as impaired renal function, atrial fibrillation, and congestive heart failure. These comorbidities are known to be associated with elevated hs-cTNT levels [8, 18].

Our data highlights the importance of interpreting hs-cTNT as an important risk predictor in patients presenting with acute dyspnea at the ED and without primary cardiac disease. Importantly, this seems to address patients with specific comorbidities such as renal impairment as well as heart failure and atrial flutter. More studies are necessary to evaluate and validate the strength of hs-cTNT in predicting mortality and other adverse events in this group of patients in order to improve risk stratification. The ability of METTS-A to identify patients with acute MI is so far not studied. Another triage system, the Manchester Triage System (MTS), has been evaluated about the sensitivity and specificity in risk prioritization of patients with acute MI with the presenting symptom of chest pain. The conclusion of this study was that MTS had only a moderate sensitivity and specificity to evaluate patients with acute MI [23]. Our study points out the importance of testing the METTS-A triage system for its ability to rule in and rule out patients with acute MI in different groups of ED admitted patients. Finally, for BMI, the lower mortality risk with increasing hs-cTNT values seems to fit with the obesity paradox, i.e. lower mortality for patients with obesity under special conditions [24].

There are limitations with this study. Firstly, this study was a observational study based on patients seeking care with dyspnea at the ED. We did not have access to a second hs-cTnT or anamnestic details (definition type 2, 10), and were thus not able to separate myocardial injury from a type 2 infarction. Even though we believe that primary MI’s were excluded by the help of the RETTS-A triage algorithm and with the additionally help of the research nurse we cannot rule out the possibility that some Type 2 myocardial infarctions still remained within the cohort.

We also had no data on whether the patients had undergone testing for cardiac diseases before and after inclusion of the study or if there was any previous contact with an cardiologist. Neither do we have data whether the patients were prescribed any preventive medicine, for example aspirin.

The strength of the study is the very carefully characterized cohort of patients admitted to ED due to acute dyspnea used in this study. All patients entered normal triage in the emergency department, with thorough clinical assessment, blood testing, clinical examination and if given, further clinical care. Data have afterwards been linked to national registries for additional information. Specifically, a strength of this study is the specific setup of this study in its effort to exclude patients with acute primary cardiac disease by the study nurses at inclusion and after in-hospital care of the patient.

In conclusion, we show that hs-cTnT levels predicted risk of 3-months mortality among patients with dyspnea at the ED without an acute coronary syndrome. Hs-cTnT seems to be an important biomarker within this patient group and elevated levels indicate a need for thorough investigation and to be tested prospectively for possible implementation in clinical guidelines.

## Electronic supplementary material

Below is the link to the electronic supplementary material.

Supplementary Material 1

## Data Availability

The datasets generated during and/or analyzed during the current study are not publicly available due to restraints in the ethical approvals but are available from the authors (Toralph Ruge or Olle Melander) on reasonable request and with approval from ethical board.

## Abbreviations

ADYS: Acute Dyspnea Study
AF: Atrial fibrillation
ANOVA: Analysis of variance
CHD: Coronary heart disease
CHF: Congestive heart failure
ECG: Electrocardiography
ED: Emergency department
GFR: Glomerular filtration rate
HR: Hazard ratio
Hs-cTnT: High sensitivity cardiac troponin T
METTS-A: Medical Emergency Triage and Treatment System-Adult score
MI: Myocardial infarction
NYHA: New York Heart Association
RETTS: Rapid emergency triage and treatment system
VTE: Venous thromboembolism
