# Supplementary material for: Myocardial injury defined as elevated high-sensitivity cardiac troponin T is associated with higher mortality in patients seeking care at emergency departments with acute dyspnea
Source: BMC Emerg Med. 2023 Apr 5;23:40. doi: 10.1186/s12873-023-00787-w (PMC10074855; doi:10.1186/s12873-023-00787-w)
Supplement: Supplementary file 1 — Supplementary Material 1 [file 12873_2023_787_MOESM1_ESM.docx]

**Supplementary Table 1.** Patient characteristics at baseline by acquired high-sensitive cardiac Troponin T (hs-cTnT) value or not

|  | hs-cTnT acquired  (n=1001) | hs-cTnT not acquired  (n=453) |
| --- | --- | --- |
| Age, years (mean, SD) | 71.0 (17.4) | 66.4 (19.6) |
| Age, years (median, IQR) | 74.8 (62.6-84.0) | 70.8 (53.0 – 81.5) |
| Men (N, %) | 470 (47.0%) | 184 (40.6%) |
| History of CAD (N, %) | 316 (31.6%) | 87 (19.2%) |
| History of CHF (N, %) | 363 (36.3%) | 97 (21.4%) |
| History of AF (N, %) | 318 (31.8%) | 101 (22.3%) |
| History of hypertension (N, %) | 443 (44.3%) | 162 (35.8%) |
| History of renal disease (N, %) | 95 (9.5%) | 39 (8.6%) |
| METTS-green (N, %) | 42 (4.2%) | 52 (11.5%) |
| METTS-yellow (N, %) | 489 (48.9%) | 259 (57.2%) |
| METTS-orange (N, %) | 334 (33.4%) | 102 (22.5%) |
| METTS-red (N, %) | 132 (13.2%) | 37 (8.2%) |
| NYHA-class I (N, %) | 302 (30.2%) | 145 (32.0%) |
| NYHA-class II (N, %) | 316 (31.6%) | 163 (36.0%) |
| NYHA-class III (N, %) | 168 (16.8%) | 58 (12.8%) |
| NYHA-class IV (N, %) | 204 (20.4%) | 73(16.1%) |
| ECG normal ST (N, %) | 455 (45.5%) | 227 (50.1%) |
| ECG ST Elevation (N, %) | 13 (1.3%) | 4 (0.9%) |
| ECG ST Depression (N, %) | 108 (10.8%) | 24 (5.3%) |
| ECG Abnormal T-wave (N,%) | 77 (7.7%) | 25 (5.5%) |
| hs-cTnT, ng/L (mean +/- SD) | 38.23 +/- 105.83 | - |
| hs-cTnT, ng/L (median, IQR) | 19.00 (8.00 – 37.50) | - |
| Ambulance (N, %) | 551 (55.0%) | 201 (44.4%) |
| Alarm (N, %) | 107 (10.7%) | 34 (7.5%) |
| Hospitalization (%) | 598 (59.7%) | 197 (43.5%) |

CAD: coronary artery disease; CHF: congestive heart failure; AF: atrial fibrillation; ED: emergency department

IQR: interquartile range
